# The function of CD164 in breast cancer and its possibility as a molecular biomarker: bioinformatics analysis and experimental validation

**DOI:** 10.3389/fimmu.2025.1601547

**Published:** 2025-07-07

**Authors:** Mengxin Li, Juanjuan Mao, Guang Wang, Jiasi Chen, Jinghui Hong, Xue Wang, Baoling Liang, Dong Song

**Affiliations:** ^1^ Department of Breast Surgery, General Surgery Center, First Hospital of Jilin University, Changchun, China; ^2^ Department of Biomedical Science, College of Basic Medical Sciences, Jilin University, Changchun, China

**Keywords:** breast cancer, molecular targeted therapy, CD164, immune microenvironment, apoptosis

## Abstract

**Background:**

Breast cancer is the most prevalent malignancy among women globally. Molecular-targeted therapy improves treatment efficacy by precisely targeting tumor-specific molecules, minimizing damage to healthy tissues. Identifying new molecular targets is essential for enhancing therapeutic outcomes and prognosis in breast cancer.

**Methods:**

The TCGA database was used to assess CD164 expression in breast cancer and its correlation with prognosis. Chemosensitivity analysis was performed to evaluate the association between CD164 and response to targeted therapies. Immune infiltration analysis was conducted to assess the relationship between CD164 expression and immune cell populations. CCK-8 assays, clonogenic assays, and flow cytometry analyses were employed to examine the effects of CD164 knockdown on cell viability, proliferation, cell cycle progression, and apoptosis. RNA sequencing and Gene Set Enrichment Analysis (GSEA) were performed to identify pathways regulated by CD164.

**Results:**

CD164 was highly expressed in breast cancer tissues and correlated with poorer prognosis, including shorter disease-free and overall survival. Chemosensitivity analysis linked CD164 to sensitivity to multiple targeted therapies, suggesting its role in oncogenic pathways. Immune infiltration analysis revealed CD164’s association with immunosuppressive cells, including resting CD4 memory T cells, M2 macrophages, and mast cells, while exhibiting a negative correlation with Tregs and NK cells, underscoring its significance in the immunosuppressive tumor microenvironment. CD164 knockdown inhibited cell viability and proliferation and induced cell cycle arrest and apoptosis. RNA sequencing and GSEA showed that CD164 regulates proliferation, metabolism, migration, and adhesion pathways while suppressing tumor-promoting pathways and activating immune-related pathways.

**Conclusions:**

CD164 plays a critical role in breast cancer progression, influencing tumor growth, immune evasion, and therapeutic response. It represents a promising therapeutic target, offering potential for improving breast cancer treatment and prognosis.

## 1 Introduction

Breast cancer is now the most common malignancy among women and the most often diagnosed cancer worldwide according to the most recent global cancer statistics published by the International Agency for Research on Cancer (IARC) of the World Health Organization (1). Treatment and diagnosis of breast cancer depend much on molecular markers (2). Hormone receptor–positive/ERBB2-negative (accounting for roughly 70% of cases), ERBB2-positive (15%–20%), and triple-negative breast cancer (15%), based on the expression of molecular markers including estrogen receptor (ER), progesterone receptor (PR), and human epidermal growth factor receptor 2 (ERBB2). Conventional approaches, including surgery combined with radiotherapy and chemotherapy, as well as molecular targeted treatments, including endocrine therapy and anti-HER2 therapy, define current treatment modalities for breast cancer.

Even with these treatments available, a subset of patients still has a bad prognosis, mostly due to multidrug resistance and side effects related to conventional treatments (2). Targeting tumor cells displaying particular molecular traits, molecular targeted treatment seeks to be selective. Targeted treatments help to more precisely manage tumors by reducing tumor cell proliferation, upsetting the cell cycle, causing death, and so suppressing angiogenesis (3, 4). Additionally, it has been demonstrated that combined targeted therapy with chemotherapy or immunotherapy lowers tumor drug resistance and enhances clinical results (5, 6). Clarifying the pathophysiology of breast cancer and identifying novel molecular targets for diagnosis and treatment are therefore imperative.

Known to control hematopoietic progeny cell migration, adhesion, and proliferation, CD164 is a transmembrane sialomucin and cell adhesion molecule (7). Previous research has shown that CD164 can interact with the CXC chemokine receptor 4 (CXCR4), so regulating muscle development (8). CD164 expression is significantly raised in patients with Sézary syndrome; mutations in this gene may be connected to hearing loss (9). Additionally, greatly overexpressed in many malignancies, including glioma (8), prostate cancer (10), colon cancer (11), and bladder cancer (12), is CD164. Silencing CD164 expression clearly reduces tumor cell growth and causes apoptosis, according to functional studies. Moreover, aiming at CD164 can improve the radiosensitivity of non-small cell lung cancer cells (13). These results taken together imply that CD164 may act as a predictor of tumor cell sensitivity to radiotherapy and chemotherapy as well as a potential molecular marker for tumor diagnosis and treatments. However, studies on the function of CD164 in breast cancer are few. Therefore, this work intends to investigate the molecular function of CD164 in this context and the correlation between CD164 expression and breast cancer.

## 2 Materials and methods

### 2.1 Data acquisition and collection

The standardized pan-cancer dataset (*N* = 19,131, *T* = 60,499) was obtained from the UCSC Xena database (https://xenabrowser.net/), from which the expression and survival data of the CD164 gene (ENSG00000135535) were extracted across various samples. The Cancer Genome Atlas (TCGA) database (https://portal.gdc.cancer.gov/) yielded RNA-sequencing data and matching clinical information for breast cancer. This dataset was used to evaluate the differential expression of CD164, including 1,092 tumor samples and 292 normal samples. Furthermore, the Kaplan–Meier Plotter Database, which combines clinical prognostic data for survival analyses with gene expression profiles, was used. An independent validation set consisted of the GSE45255 dataset taken from the Gene Expression Omnibus (GEO) database (https://www.ncbi.nlm.nih.gov/geo/).

### 2.2 Analysis of drug sensitivity

We used pharmacogenomic data from the Genomics of Drug Sensitivity in Cancer (GDSC) database (https://www.cancerrxgene.org/) and the “oncoPredict” R software package to project the chemosensitivity of tumor samples. We predicted the IC50 for every chemotherapeutic agent over tumor samples using regression-based modeling. Tenfold cross-validation on the GDSC training set validated model robustness and predictive accuracy. Including the computation of average expression levels for duplicated genes and the application of the “combat” technique to adjust for batch effects, all settings were kept at default.

### 2.3 Gene Set Variation Analysis

The non-parametric, unsupervised Gene Set Variation Analysis (GSVA) method is used to figure out which gene sets are more common in transcriptomic data. GSVA lets gene-level expression changes be translated into functional insights at the pathway level by scoring gene sets holistically. Gene sets were downloaded from the Molecular Signatures Database and subsequently scored in this work using the GSVA method. This scoring system sought to find and describe possible functional changes in biological processes among the samples investigated.

### 2.4 Gene Set Enrichment Analysis

One often used approach for clarifying the complex interaction between disease classification and biological relevance is Gene Set Enrichment Analysis (GSEA). High and low CD164 expression groups were formed from the patients, and GSEA was applied to examine the variations in signaling pathways among them. Version 7.0 of the MsigDB database produced annotated gene sets for pathway subtypes, so it served as the reference gene set used for the analysis. A differential expression analysis was used to compare pathway activities among the subtypes; gene sets that were significantly enriched (with adjusted *p*-values less than 0.05) were ranked according to their consistency scores.

### 2.5 Analysis of immune cell infiltration

Using a support vector regression-based deconvolution method, CIBERSORT, immune cell composition was deduced from breast cancer RNA-sequencing data. The method looked for links between CD164 expression and immune cell infiltration and got a rough idea of the ratios of 22 different types of immune cells in the area around the tumor. Considered statistically significant only were findings with *p*-values<0.05.

### 2.6 Cell culture

The Chinese Academy of Sciences’ Shanghai Institute of Cell Biology provided the SKBR3, MCF7, and 4T1 cell lines for breast cancer. The cells were cultured in high-glucose DMEM (catalog number RNBM1891, Sigma, GB) with 1% penicillin/streptomycin, 1% sodium pyruvate, and 10% fetal bovine serum (Gibco, USA) added as supplements. For the next experiments, all the cell lines were kept at 37°C in an incubator that was humidified and had 5% CO_2_.

### 2.7 Cell viability

Seeded into 96-well plates, SKBR3 and MCF7 cells (4 × 10^4^ cells/well) were incubated at 37°C with 5% CO_2_ for 24h. Lipofectamine 3000 transfected small interfering RNA (siRNA) targeting CD164 into cells for 48h. The CCK-8 assay (MedChemExpress, Monmouth Junction, NJ, USA) was used to evaluate cell proliferation; optical density (OD) was measured at 450 nm with a spectrophotometer (Bio-Rad Laboratories, Inc., USA).

### 2.8 Reagents

Purchased from R&D Systems, Minneapolis, MN, USA, the human CD164 antibody (1:2000, cat. no. AF5790-SP). ProteinTech Group, Inc., Rosemont, IL, USA, supplied bax (1:2000, cat. number 50599-2-Ig), bcl2 (1:2000, cat. number 12789-1-AP), caspase 3/p17/p19 (1:2000, cat. number 19677-1-AP), and actin antibodies. ProteinTech Group, Inc., Rosemont, LA, USA, supplied secondary antibodies horseradish peroxidase (HRP)-conjugated anti-mouse IgG (H+L) (1:5000, cat. no. SA00001-1) and anti-rabbit IgG (H+L). GenePharma (Shanghai, China) designed siRNAs targeting the CD164 genes. The sequences of the siRNAs used were as follows:

siCD164-1(5′-CCCGAACGUGACGACUUUA-3′).
siCD164-2(5′GGACUGGUGAUUCAUUUGU-3′).
siCd164-1(5′-AGCUGUGUUUCCUGUGUUAAU-3′).
siCd164-2(5′-GACCUAUUGUGCAAAUGAACC-3′).

### 2.9 Colony-forming cell (CFC) assay, immunohistochemistry, cell cycle, cell apoptosis, and Western blotting analysis

Colony-forming cell (CFC) assay, immunohistochemistry, cell cycle analysis, cell death assay, and Western blotting were carried out as previously described (14).

### 2.10 Animal experiments

Beijing Vital River Laboratory Animal Technology supplied female Balb/c and Balb/c nude mice. Approved by the Scientific Investigation Board of the First Hospital of Jilin University College of Translational Medicine (Changchun, China), all animal experiments followed the NIH Guide for the Care and Use of Laboratory Animals. One × 10^5^ SKBR3 cells were subcutaneously injected into the flanks of nude mice, while five × 10^5^ 4T1 cells were injected into the mammary fat pads of Balb/c mice. After tumor implantation, mice were randomly assigned to one of two groups (*n* = 5) (1): siNC (negative control) treatment or (2) siCD164 treatment. GenePharma-synthesized siRNA-CD164 with 2’-O-Me modifications (0.5 mg/kg) was intratumorally injected every 48 hours, with modified scramble siRNA serving as the negative control. Every three days, we took measurements of the tumor’s size and weight. After 21 days, the mice were put to sleep, and tumor samples were taken for immunohistochemical (IHC) and Western blotting analyses.

### 2.11 Statistical analysis

The standard deviation was displayed alongside the average data from a minimum of three replicates. For statistical analyses, Microsoft Excel and GraphPad Prism v6.00 were utilized. For pairwise comparisons, an unpaired Student’s t-test was employed; for multiple comparisons, a one-way ANOVA in conjunction with a Newman–Keuls *post-hoc* test was employed. R language (version 4.3.0) was used for all statistical tests, with a *p*-value of 0.05 for statistical relevance.

## 3 Results

### 3.1 CD164 is highly expressed in breast cancer and correlates with prognosis

**Figure 1:**
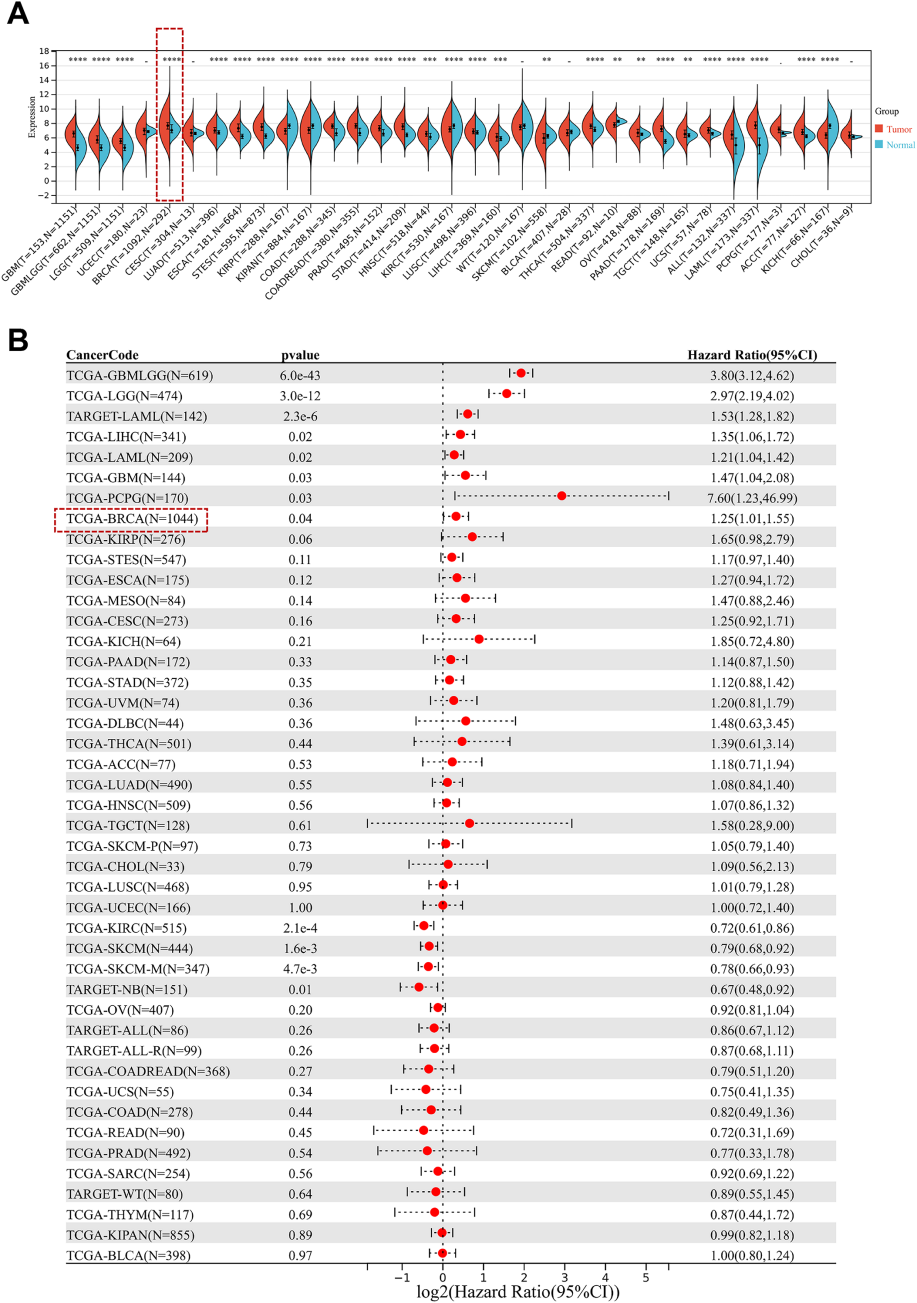
Prognostic relevance of CD164 expression in diverse cancer types. **(A)** CD164 expression levels in pan-cancer tissues versus adjacent normal tissues. Data was obtained from the UCSC Xena database. **(B)** Forest plot demonstrating the prognostic relevance of CD164 expression across various cancer types, featuring hazard ratios (HR) and 95% confidence intervals (CI) that reflect its correlation with overall survival. Data was obtained from the TCGA and TARGET databases. *p < 0.05, **p < 0.01, ***p < 0.001, ****p < 0.0001.

Analysis of mRNA expression data across multiple cancer types using the Pan-Cancer database revealed that tissue of breast cancer has CD164 greatly overexpressed (**Figure 1A**). Additionally, we found that elevated CD164 expression was strongly associated with poorer prognosis in eight tumor types, including glioblastoma, lower grade glioma, acute myeloid leukemia from the TARGET dataset, breast cancer, liver hepatocellular carcinoma, acute myeloid leukemia, and pancreatic cancer (**Figure 1B**), emphasizing its potential as a prognostic marker and therapeutic target in various cancers.

**Figure 2:**
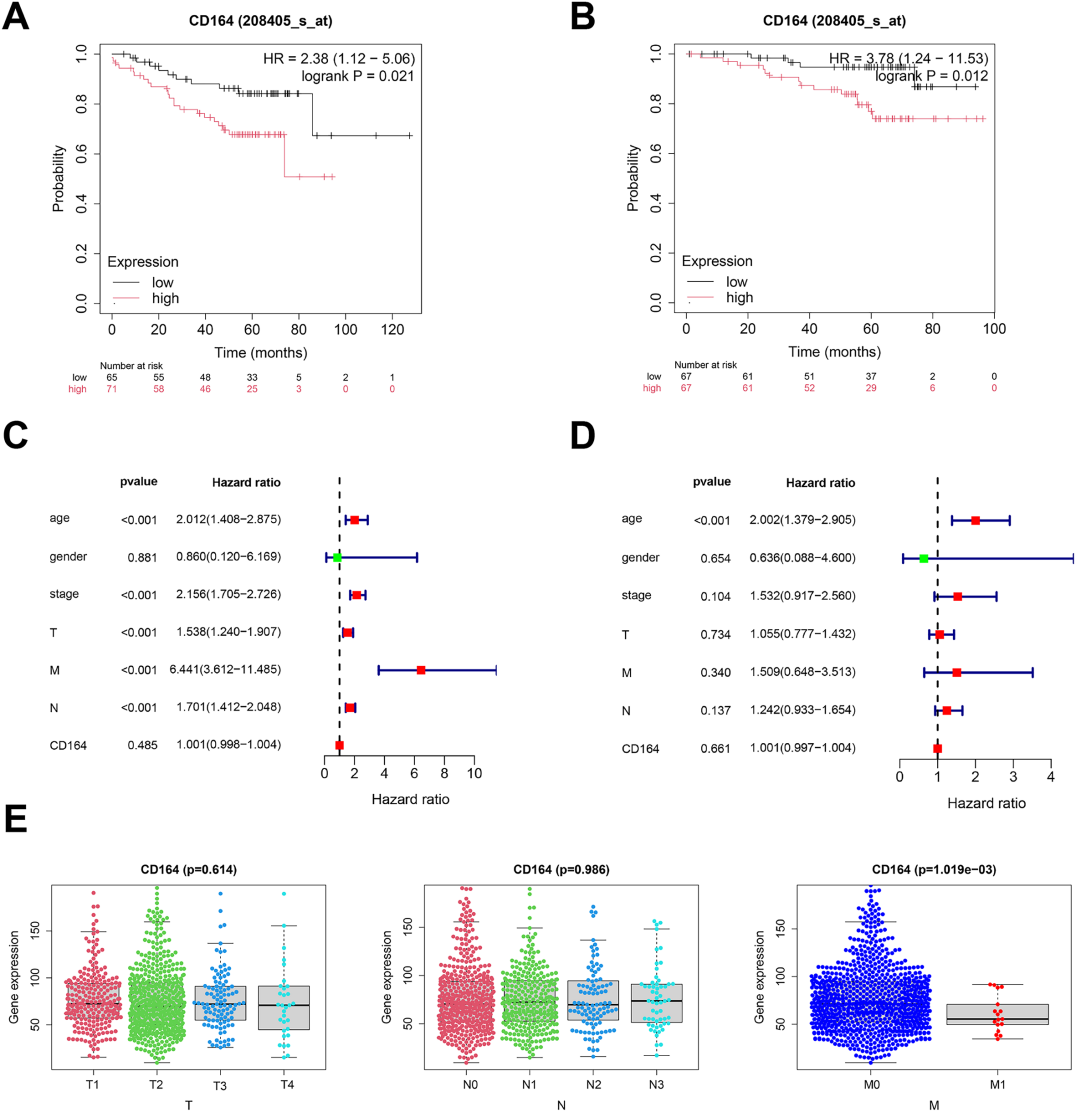
Prognostic relevance of CD164 expression in breast carcinoma. **(A)** DFS assessed via the Kaplan–Meier method. Data acquired from the GEO database (*p* = 0.021). **(B)** OS assessed utilizing the Kaplan–Meier method. Data acquired from the GEO database (*p* = 0.012). **(C)** Univariate and **(D)** multivariate Cox regression analyses of clinical factors in conjunction with CD164 expression. **(E)** CD164 expression throughout TNM stages in breast cancer. Data was obtained from the TCGA database.

At the optimal cutoff point, survival analysis depending on expression levels revealed a noteworthy correlation between CD164 expression and BC patient survival. Compared to patients with low expression, those who had high levels of CD164 had much shorter disease-free survival (**Figure 2A**) and overall survival (**Figure 2B**); a significant inverse relationship between CD164 expression and patient prognosis was found by Kaplan–Meier Plotter survival analysis. Using clinical data and CD164 expression data, univariate and multivariate Cox regression models were built; related forest plots were produced (**Figures 2C, D**). Further analyses revealed a strong correlation between the M stage of breast cancer patients and CD164 expression levels (**Figure 2E**). When combined, these findings suggest that CD164 may be a biomarker for the detection, management, and prognostic assessment of breast cancer.

### 3.2 The connection between drug sensitivity and CD164 expression

**Figure 3:**
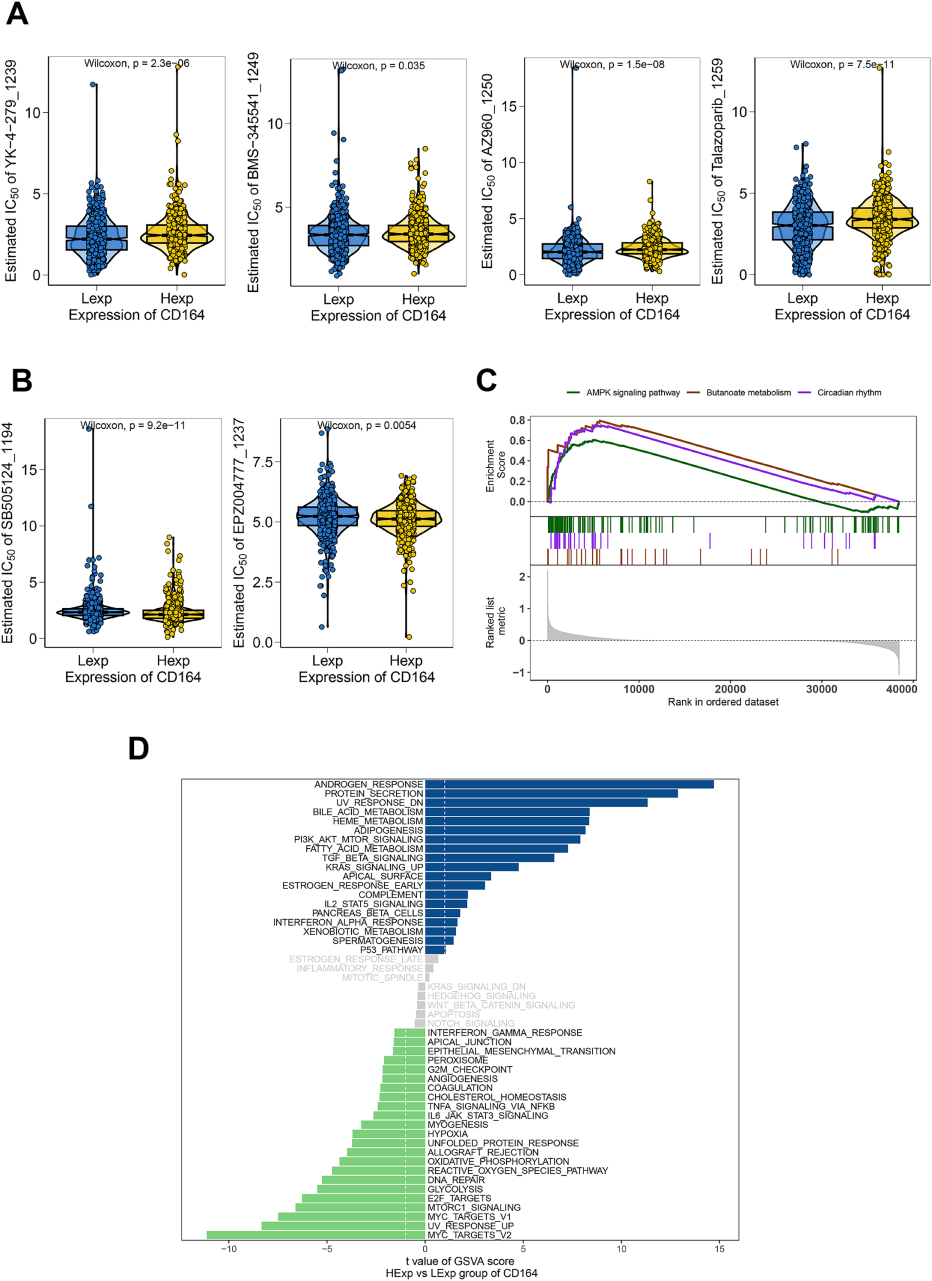
Chemosensitivity and functional enrichment analyses of CD164 in breast carcinoma. **(A)** Chemosensitivity analysis of breast cancer specimens utilizing drug response data from the GDSC database. The pharmacological sensitivity profiles of four targeted agents—YK-4-279 (RNA helicase A inhibitor), BMS-345541 (IKK inhibitor), AZ960 (JAK2/3 inhibitor), and Talazoparib (PARP inhibitor)—were stratified by CD164 expression status (Low Expression [Lexp] vs. High Expression [Hexp]). Violin plot distributions revealed consistently lower IC50 values (indicating greater sensitivity) in CD164-low tumors across above compounds. **(B)** The pharmacological sensitivity profiles of two targeted agents—SB505124 (TGF-β receptor inhibitor) and EPZ004777 (DOT1L inhibitor)—were also stratified by CD164 expression status (Lexp vs. Hexp). Violin plot distributions revealed consistently lower IC50 values (indicating greater sensitivity) in CD164-high tumors across above compounds. **(C)** GSEA analysis revealed CD164’s involvement in regulating the AMPK signaling pathway, Butanoate metabolism, and Circadian rhythm pathways. **(D)** GSVA analysis was performed to investigate the molecular mechanisms through which CD164 facilitates tumor progression. The gene sets were ranked based on the GSVA scores. Data was acquired from the Molecular Signatures Database.

Analyzing drug response data from the GDSC database in early breast cancer samples allowed us to evaluate the possible function of CD164 in modulating chemosensitivity. The results demonstrated that CD164 expression was significantly correlated with the sensitivity to several compounds, including YK-4-279 (targeting the EWS-FLI1 fusion protein), BMS-345541 (targeting the NF-κB signaling pathway), AZ960 (targeting the JAK2/3 signaling pathway), Talazoparib (targeting PARP inhibition), SB505124 (targeting the TGF-β signaling pathway), and EPZ004777 (targeting DOT1L) (**Figures 3A, B**). Specifically, YK-4-279, BMS-345541, AZ960, and Talazoparib showed a negative correlation with CD164 expression, where CD164 low expression (Lexp) corresponded to lower IC50 values (indicating higher sensitivity). In contrast, SB505124 and EPZ004777 exhibited a positive correlation, where CD164 high expression (Hexp) was associated with lower IC50 values (indicating higher sensitivity). These results imply that CD164 might help to control the specified signaling channels.

GSVA and GSEA enrichment studies were carried out to investigate the molecular pathways by which CD164 affects tumor development even more. Results of GSVA revealed notable enrichment of androgen response and protein secretion pathways in the CD164 high-expression group (**Figure 3D**). Furthermore, well-known as traditional indicators of endocrine resistance, androgens (15) are known to control cell proliferation and death. In addition, greatly enriched in the high-CD164 expression group were important oncogenic pathways linked with proliferation and drug resistance, including PI3K-Akt, TGF-β, and IL2-STAT5. On the other hand, the low-CD164 expression group revealed rather enrichment in pathways linked to cell cycle control and DNA repair, including E2F targets and MYC targets. Further closely related to drug metabolism, excretion, and pharmacodynamics, GSEA analysis also revealed that CD164 modulates pathways, including AMPK signaling, butyrate metabolism, and circadian rhythm, so influencing tumor progression (**Figure 3C**).

In conclusion, CD164 might be a biomarker for estimating the sensitivity of chemotherapy for breast cancer. Aiming for CD164 could provide a fresh approach for tailored treatment plans for breast cancer patients.

### 3.3 The interconnection of CD164 expression and immune microenvironment

**Figure 4:**
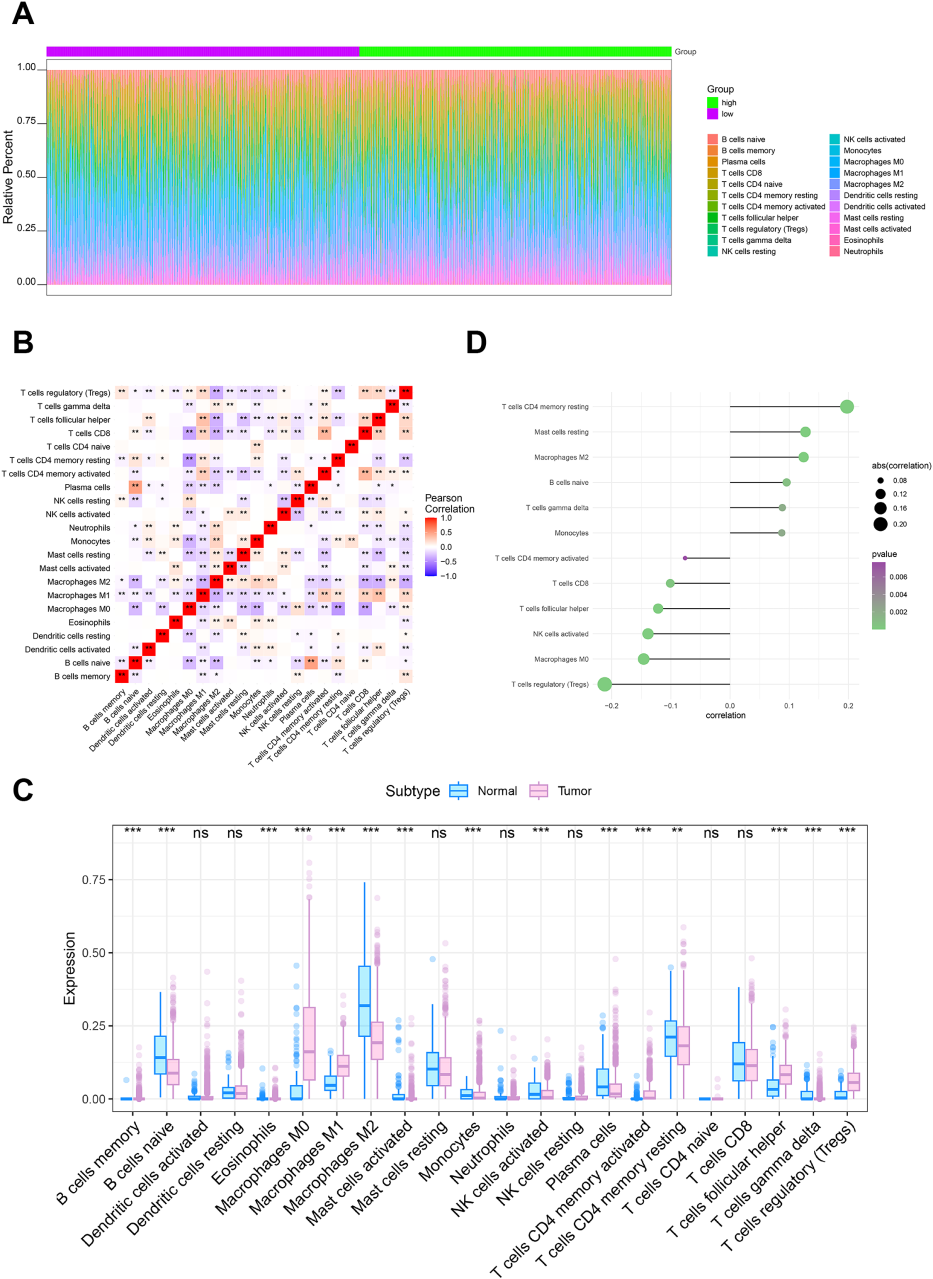
Relationship between CD164 expression and the tumor immune microenvironment in breast cancer. **(A, B)** Analysis of immune cell infiltration in breast cancer specimens from the TCGA dataset. **(C)** Relationship between CD164 expression levels and immune cell infiltration profiles. **(D)** Comparative analysis of immune cell expression in breast tumor tissues versus adjacent normal tissues. *p < 0.05, **p < 0.01, ***p < 0.001. ns indicates no significance.

The microenvironment around a tumor is made up of immune cells, growth factors, inflammatory factors, cancer cells, and extracellular matrix components, all of which have a fundamental impact on tumor diagnosis, survival outcomes, and therapeutic sensitivity (16, 17). We investigated the link between CD164 expression and immune cell infiltration by TCGA dataset analysis. Our results showed differences in immune cell ratios among patients and relationships among several immune cell populations (**Figures 4A, B**). Especially notable variations in immune cell populations between tumor and normal tissue groups included memory B cells, naive B cells, and eosinophils (**Figure 4D**). We then looked closer at the link between CD164 and immune cells. Suggesting that CD164 may help to control the immunosuppressive microenvironment, the analysis revealed a notable positive correlation between CD164 and resting CD4 memory T cells, M2 macrophages, and resting mast cells. By focusing on CD164, immunotherapy’s efficacy may be raised, and the immune system’s reaction to cancer could be strengthened (**Figure 4C**). On the other hand, CD164 expression was considerably negatively linked with regulatory T cells (Tregs), M0 macrophages, and activated natural killer (NK) cells. These findings suggest that CD164 may function as a multifaceted immune regulator, where its inhibition could (1) alleviate Treg-mediated immunosuppression while (2) dynamically reshaping the innate immune landscape through modulation of M0 macrophage polarization and NK-cell activity. Such coordinated immunomodulatory effects may collectively foster a tumor microenvironment more receptive to immunotherapeutic interventions (**Figure 4C**).

### 3.4 Silencing of CD164 impedes proliferation and triggers apoptosis in breast cancer cells

**Figure 5:**
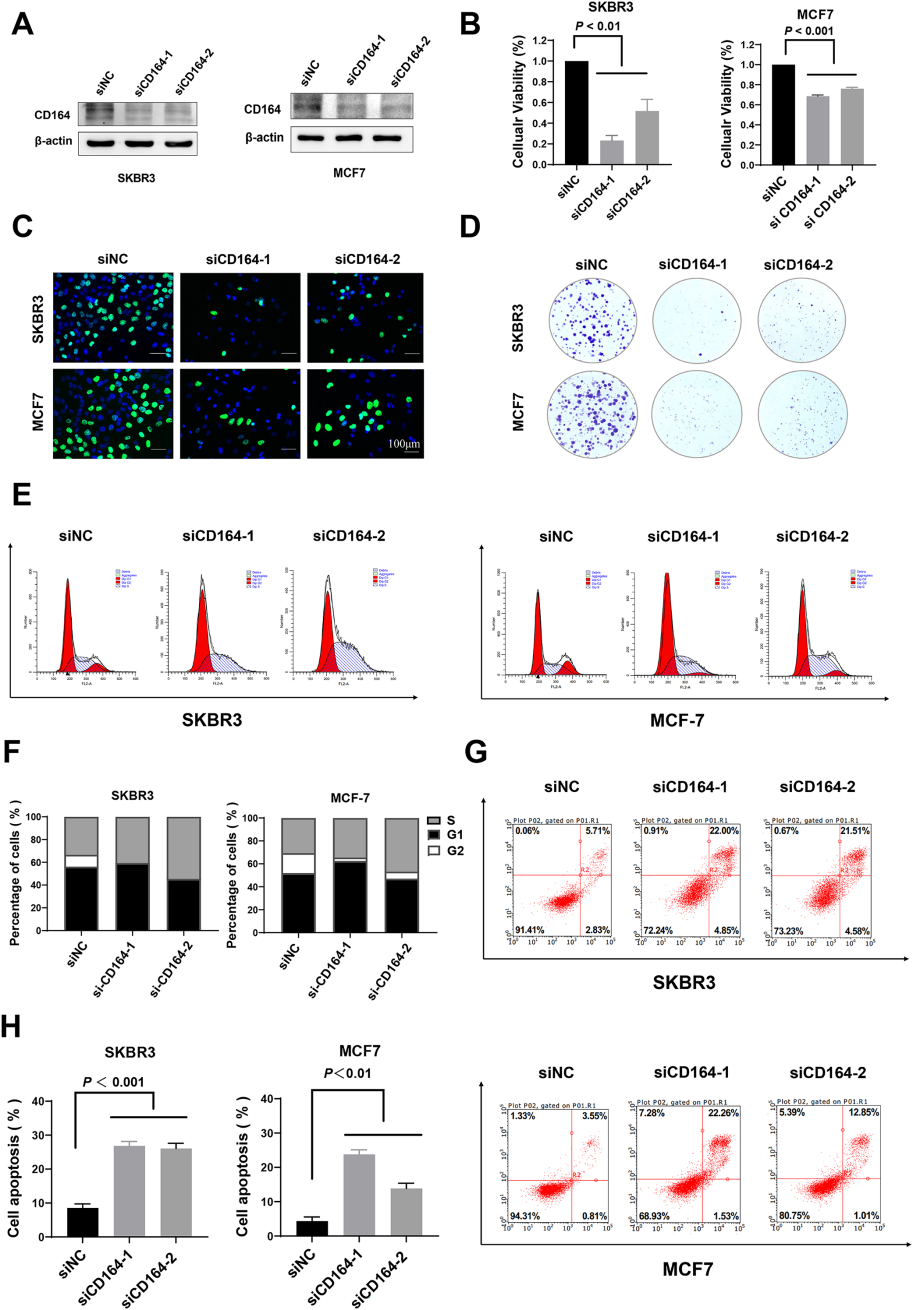
CD164 knockdown suppresses proliferation and induces apoptosis in breast cancer cells. **(A)** Western blot analysis evaluating the knockdown efficacy of siRNA-CD164 in MCF7 and SKBR3 cell lines. **(B)** Cell viability assessed via CCK-8 assay subsequent to CD164 knockdown. **(C)** EDU incorporation assay and **(D)** clonogenic assay employed to evaluate cell proliferation. **(E, F)** Flow cytometry analysis of cell cycle distribution subsequent to CD164 inhibition. Annexin V/PI staining **(G, H)** to assess apoptosis levels. For flow cytometry analysis of apoptosis, quadrants were defined as follows: LL (Annexin V^-^/PI^-^) = viable cells; LR (Annexin V^+^/PI^-^) = early apoptotic cells; UR (Annexin V^+^/PI^+^) = late apoptotic/necrotic cells; UL (Annexin V^-^/PI^+^) = necrotic/mechanically damaged cells. Quadrant boundaries were established using unstained and single-stained controls. Data are expressed as mean ± standard deviation (SD). Statistical significance was evaluated through one-way ANOVA.

MCF7 is a hormone receptor–positive (ER+, PR+) breast cancer cell line commonly used in research on hormone-sensitive breast cancer. SKBR3 is a HER2-positive breast cancer cell line that overexpresses the HER2 receptor, making it a model for HER2-targeted therapies. In this study, we utilized these two breast cancer cell lines to investigate the biological function of CD164. Two specific small interfering RNAs that target CD164 were created to find out what role CD164 plays in these two types of breast cancer cells. Western blotting (**Figure 5A**) showed that siRNA-CD164 effectively knocked down MCF7 and SKBR3 breast cancer cell lines. Then, a set of functional tests were carried out to evaluate cell viability and proliferation under CD164 inhibition. After CD164 knockdown, CCK-8 assays found a notable drop in cell viability (**Figure 5B**). Furthermore, shown by EDU incorporation assays (**Figure 5C**) and clonogenic assays (**Figure 5D**) was a rather reduced proliferative capacity of breast cancer cells.

We investigated how CD164 affects the cell cycle and death in more detail using flow cytometry. **Figures 5E, F** show how knocking down CD164 stops breast cancer cells in the S-phase of the cell cycle. In addition, Annexin V/PI staining showed a clear rise in death after si-CD164 treatment as opposed to the si-NC group (**Figures 5G, H**). However, CD164 knockdown showed no significant differences in functional phenotypes between the two breast cancer cell lines. These results highlight the important function of CD164 in controlling breast cancer cell proliferation and survival, suggesting that CD164 might be a suitable therapeutic target.

### 3.5 Transcriptomic analysis of CD164 in the modulation of biological functions in breast cancer cells

**Figure 6:**
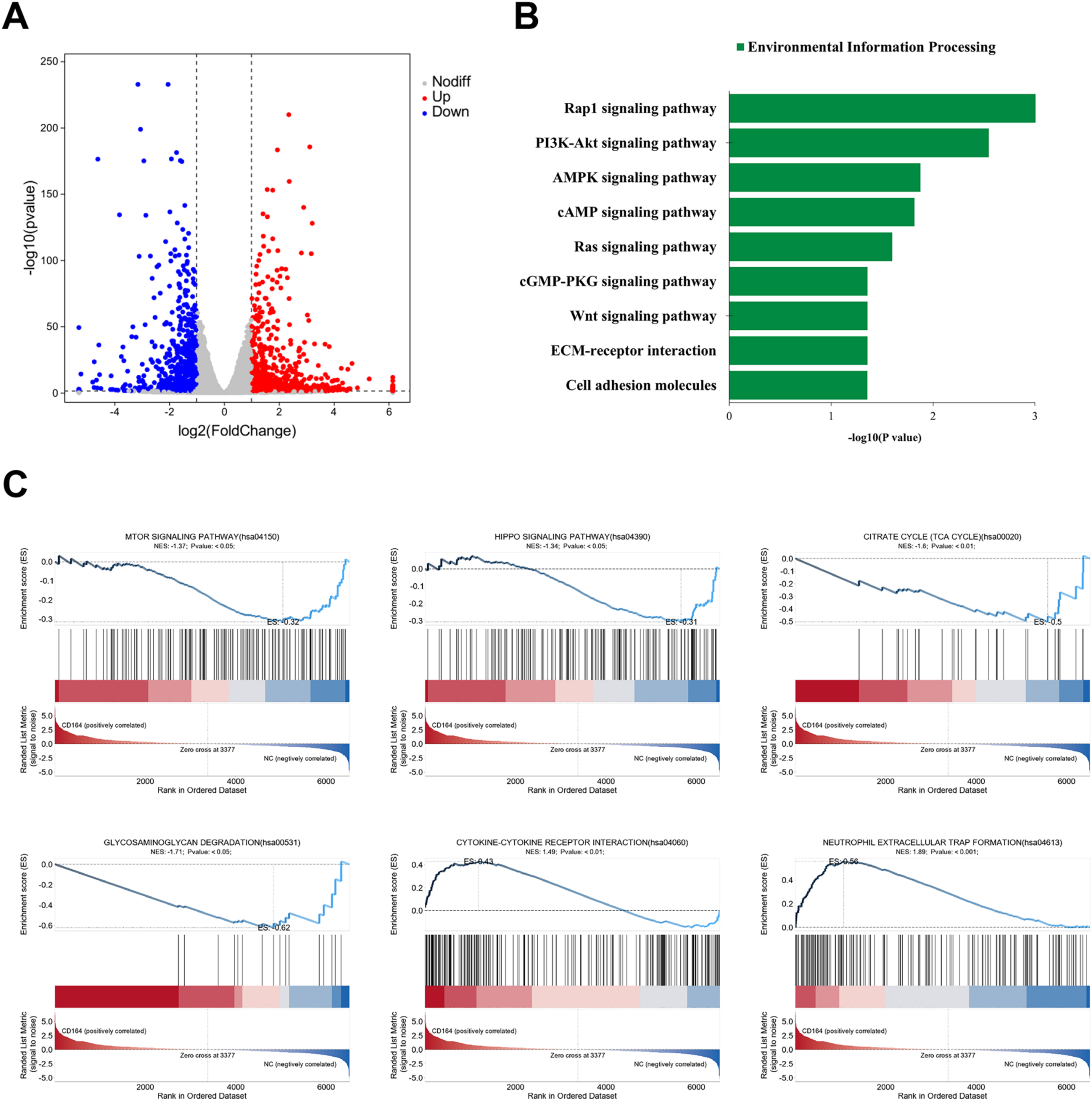
Transcriptomic analysis subsequent to CD164 knockdown in breast cancer cells. **(A)** volcano plot illustrating differentially expressed genes (865 upregulated and 732 downregulated) in the si-CD164 group relative to the si-NC control group. **(B)** KEGG pathway enrichment analysis emphasizing notable changes in pathways associated with proliferation, metabolism, migration, and adhesion. **(C)** GSEA demonstrating both the inhibition and activation of critical signaling pathways subsequent to CD164 suppression. Data are expressed as mean ± SD. Statistical significance was evaluated through one-way ANOVA.

RNA sequencing was conducted to clarify the CD164 regulatory systems in breast cancer cell proliferation even more. CD164 knockdown produced at the transcriptional level downregulation of 732 genes and upregulation of 865 genes when compared to the si-NC group (**Figure 6A**). Under the category of “Environmental Information Processing,” KEGG pathway enrichment analysis revealed that CD164 knockdown considerably changed pathways involved in proliferation (PI3K/AKT, cAMP, Ras, Wnt), metabolism (AMPK signaling), migration (Rap1, cGMP-PKG), and adhesion (cell adhesion molecules, ECM-receptor interaction) (**Figure 6B**). All of which are related to cell proliferation, metabolism, and extracellular matrix stiffness, GSEA analysis also revealed that mTOR (Normalized Enrichment Score [NES] = −1.37), HIPPO (NES = −1.34), TCA cycle (NES = −1.6), and glycosaminoglycan degradation (NES = −1.7) pathways were significantly inhibited. Pathways activated in contrast, suggesting increased anti-tumor immune responses, were cytokine-cytokine receptor interaction (NES = 1.49) and neutrophil extracellular trap development (NES = 1.89). **Figure 6C** These findings taken together emphasize the complex function of CD164 in controlling important oncogenic signaling pathways, so supporting its possible use as a diagnostic and therapeutic target in breast cancer.

### 3.6 Downregulation of CD164 inhibits breast cancer progression *in vivo*

**Figure 7:**
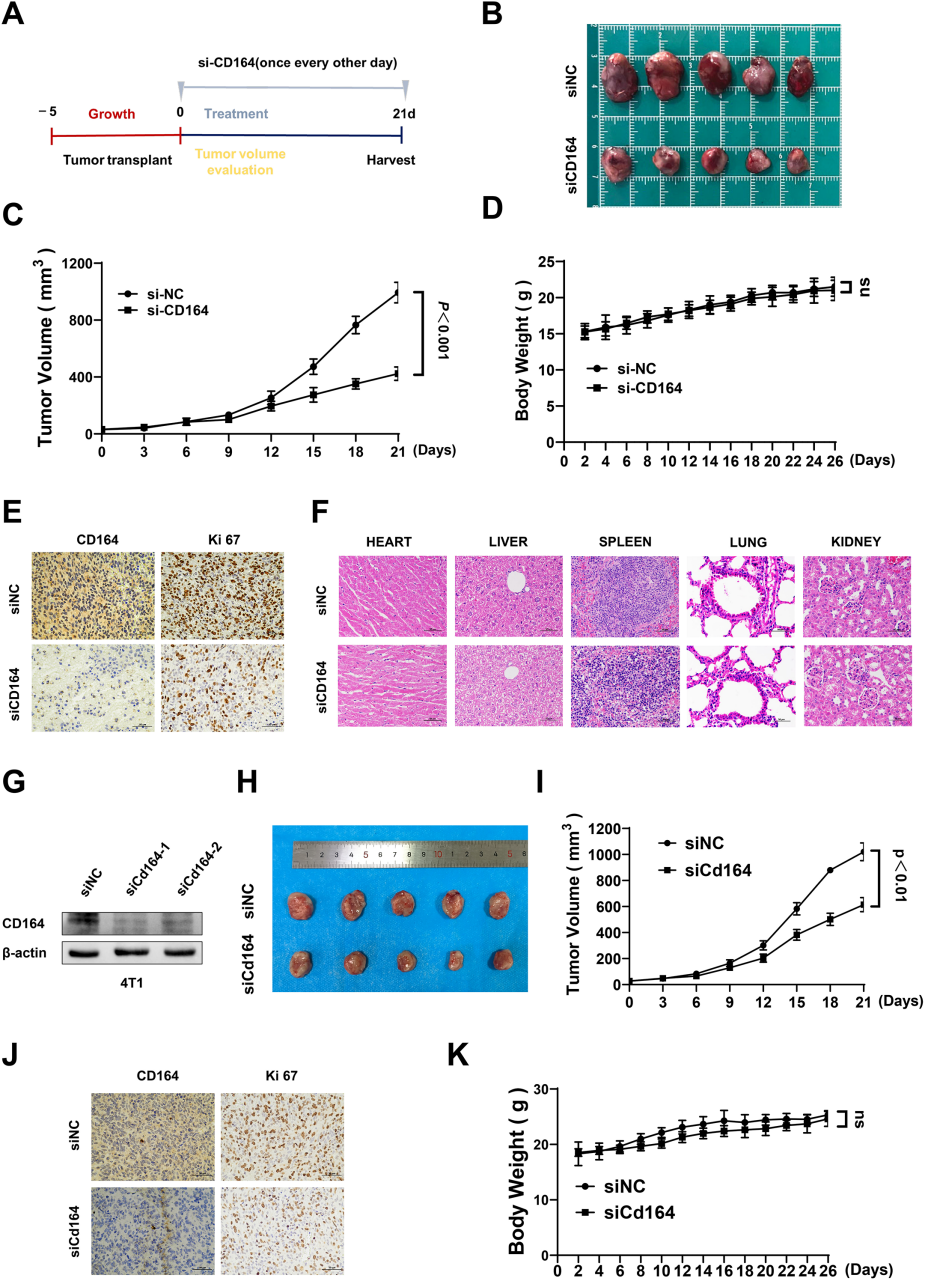
The downregulation of CD164 impedes breast cancer progression *in vivo*. **(A)** Schematic representation of the tumor implantation and therapeutic protocol. **(B)** Measurement of tumor volume and **(C)** assessment of tumor growth rates comparing the si-CD164 treatment group to the control group throughout the treatment period in nude mice. **(D)** Monitoring alterations in body weight of nude mice during the treatment duration. **(E)** Immunohistochemical staining of tumor tissues for Ki67, a marker of proliferation. **(F)** Histological analysis of primary internal organs to evaluate potential toxicity (scale bars = 50 μm). **(G)** Western blot analysis evaluating the knockdown efficiency of siRNA-Cd164 in the 4T1 cell line. **(H)** Measurement of tumor volume and **(I)** assessment of tumor growth rates comparing the si-Cd164 treatment group to the control group throughout the treatment period in Balb/c mice. **(J)** Immunohistochemical staining of tumor tissues for Ki67 and CD164 (scale bars = 50 μm). **(K)** Monitoring alterations in body weight of Balb/c mice during the treatment duration. Data are expressed as mean ± SD. Statistical significance was assessed utilizing two-way ANOVA and t-tests.

Female nude mice were given subcutaneous injections of SKBR3 cells to create a xenograft model of breast cancer, so validating the biological role of CD164 *in vivo*. **Figure 7A** shows the treatment plan and experimental chronology. With a clear deceleration in tumor development seen over the treatment period, tumor volume in the si-CD164-treated group was much lower relative to the control group (**Figures 7B, C**). Immunohistochemical staining verified that the expression of the proliferation marker Ki67 in tumor tissues was much lowered by CD164 knockdown (**Figure 7E**). Significantly, mice treated with si-CD164 showed neither any appreciable change in body weight (**Figure 7D**) nor evidence of toxicity in internal organs (**Figure 7F**). To further validate the findings, we also conducted experiments using the 4T1 cell line to establish xenografts in immunocompetent Balb/c mice. Western blotting confirmed that siRNA effectively knocked down CD164 in 4T1 breast cancer cells (**Figure 7G**). Based on the highest knockdown efficiency, we selected the siCd164–1 sequence for the subsequent animal experiments. Similar to the results obtained from nude mice, CD164 knockdown in Balb/c mice also significantly suppressed tumor growth (**Figures 7H–J**). No appreciable changes in body weight were observed (**Figure 7K**). These findings show that *in-vivo* progress of breast cancer is sufficiently suppressed by CD164 knockdown.

## 4 Discussion

Treatment for breast cancer is growing more customized based on molecular biomarker expression (1). Although they seek to eradicate fast-dividing cancer cells, traditional therapeutic approaches, including surgical resections followed by adjuvant chemotherapy, sometimes cause major side effects (2, 16). Molecular targeted treatments, on the other hand, concentrate on tumor-specific biomarkers and stop tumor cell proliferation and metastases by altering important signaling pathways (4, 6). While lowering collateral damage to normal cells, this focused approach improves treatment accuracy. This means that finding new molecular therapeutic targets is important for improving early detection and treatment outcomes in breast cancer.

This work explored the function of CD164 in breast cancer by means of bioinformatics analyses. Pan-cancer analysis established CD164 as both a prognostic marker across eight malignancies and specifically in breast cancer, where high expression correlated with significantly worse disease-free survival (DFS) and overall survival (OS). The consistent association with advanced M stage further supports its clinical relevance. Notably, the drug sensitivity analysis revealed CD164’s unique association with targeted agents such as Talazoparib (PARP inhibitor) and pathway modulators (e.g., NF-κB/TGF inhibitors), suggesting its potential as a predictive biomarker for precision therapy selection. The comparative analysis of CD164 high-expression (Hexp) and low-expression (Lexp) groups in drug sensitivity assessment revealed CD164’s significant impact on chemotherapy response, underscoring its clinical relevance for treatment strategy optimization. The pathway analyses particularly demonstrated CD164’s dual role in promoting oncogenic signaling (PI3K-Akt, TGF-β) while suppressing tumor-suppressive metabolic pathways (AMPK, butyrate metabolism), providing mechanistic insight into its pro-tumorigenic effects. To sum up, data from the GDSC database then showed that CD164 modulates chemotherapy sensitivity, highlighting its possible diagnostic and therapeutic biomarker value. Moreover, GSEA and GSVA indicated that CD164 controls signaling pathways related to proliferation, metabolism, migration, and adhesion, so affecting drug resistance. The CIBERSORT algorithm was applied to deconvolve RNA-seq data from breast cancer patients, quantifying the relative proportions of 22 immune cell subtypes. Subsequent correlation analysis between gene expression levels and immune cell abundances provided preliminary insights into CD164’s potential regulatory role in the tumor immune microenvironment. Furthermore, inhibited by downregulation of CD164 expression was breast cancer cell proliferation and activity; it also caused cell cycle arrest and encouraged death. These results taken together suggest that CD164 might be a suitable target for prognosis monitoring, treatment, and diagnosis of breast cancer.

The bioinformatics analysis of the TCGA database for our study showed a strong negative correlation between the amount of CD164 expression and the number of regulatory T cells (Tregs), M0 macrophages, and activated natural killer (NK) cells. These findings suggest that CD164 may play a multifaceted role in immune regulation, potentially reducing immunosuppression through Tregs while also influencing the composition and function of other immune cell populations, including M0 macrophages and NK cells, within the tumor microenvironment. Transcriptomic sequencing data supporting this result showed that CD164 inhibition activates several immune-related pathways, including neutrophil extracellular trap formation and interactions between cytokine-cytokine receptors, so improving the anti-tumor immune response. Particularly, whereas the development of neutrophil extracellular traps helps to efficiently clear tumor cells, the activation of cytokine-cytokine receptor interactions may promote enhanced immune cell recruitment and participation in tumor immune surveillance. These findings taken together offer a viable path for immunotherapy and highlight the potential of CD164 as a new immunomodulating target. Modulating the immune system helps immunotherapy to become a therapeutic tool that improves tumor cell recognition and clearance (18). Among the main immunotherapeutic treatments are immune cell-based therapies, cancer vaccines, and immune checkpoint inhibitors (ICIs) (19). Immune checkpoint inhibitors (ICIs) restore T-cell function and enhance anti-tumor immunity by blocking immune checkpoints such as PD-1, PD-L1, and CTLA-4 (17). While immune cell therapy entails the expansion and activation of immune cells, such as T cells and NK cells, or the application of genetically modified cells, including CAR-T cells, tumor vaccines stimulate immune recognition of breast cancer cells (20). CAR-T cell therapy has shown great promise in treating blood cancers, but it is still hard to see how it can be used to treat solid tumors like breast cancer (21, 22). In recent years, research aimed at enhancing therapeutic efficacy has focused on combining immunotherapy with other modalities, including chemotherapy, targeted therapy, and radiotherapy (6, 23). Combining ICIs with HER2-targeted treatments may improve outcomes in HER2-positive breast cancer patients; evidence points to immunotherapy perhaps lowering tumor immune escape following chemotherapy (24). However, immunotherapy has immune-related side effects, including pneumonitis and hepatitis, and drug resistance remains a major obstacle to long-term effectiveness. Thus, a potential approach to improve cancer treatment is the identification of new therapeutic targets that not only stop breast cancer cell proliferation but also modulate immune responses to lower immune escape.

In this study, we investigated the functional relevance of siRNA-mediated knockdown of CD164 in breast cancer cells by means of CCK-8, EdU incorporation, and colony development assays, so evaluating cell viability, proliferation, and clonogenicities. The findings showed that in breast cancer cells, CD164 silencing greatly lowered both cell viability and proliferative capacity. The mechanisms underlying CD164’s regulatory role in cell proliferation were clarified by using flow cytometry to compare changes in cell cycle progression and death before and after CD164 knockdown. The findings demonstrated that CD164 silencing resulted in cell cycle arrest at the S phase and markedly increased the number of dead cells in the knockdown group. Together, these findings demonstrate that CD164 inhibition has a potent anti-tumor effect by causing both cell cycle arrest and death in breast cancer cells. KEGG enrichment analysis demonstrated that CD164 inhibition modulates key signaling pathways linked with cell proliferation, metabolism, migration, and adhesion. These findings suggest that CD164 may exert its effects through modulation of important signaling pathways, such as the PI3K/AKT pathway (25), which regulates cell survival and proliferation, and its dysregulation in breast cancer leads to uncontrolled growth. The cAMP pathway (26), which regulates cellular processes such as metabolism and migration, is critical for cancer progression. The Ras pathway, which is essential for regulating cell growth and survival (27), with Ras mutations often linked to aggressive tumor behavior. Aberrant Wnt signaling has been implicated in breast cancer metastasis (28), and our findings suggest CD164 may modulate this pathway to influence cell proliferation. The AMPK pathway regulates cellular energy balance and metabolic adaptation in cancer cells (29), which are key to supporting rapid cell growth. Both the Rap1 and cGMP-PKG pathways are involved in regulating cell migration and adhesion (30, 31), processes critical for tumor metastasis. Finally, disruption of cell adhesion molecules (CAMs) and ECM-receptor interactions allows cancer cells to detach and migrate, a key step in metastasis (32, 33).

Mostly found on primitive CD34+ hematopoietic progenitor cells, CD164 is a new 80–100 kDa type I transmembrane sialomucin that controls adhesion, proliferation, and differentiation (34, 35). Recent research has shown that CD164 is essential for hematopoiesis (36) as well as for the growth and invasion of malignant tumors, so promoting tumor progress (37–39). Additionally, CD164 can be used to diagnose anaphylaxis and acute lymphoblastic leukemia (40). Though the link between CD164 and cancer stem cells is still poorly known, new data points point to their possible therapeutic target, especially in HER2-positive breast cancer subtypes (41, 42). Significant overexpression of CD164 in SKBR3 cells was found by transcriptomic studies comparing the SKBR3 and MDA-MB-231 breast cancer cell lines, so suggesting its function in the course of breast cancer (41). Highly expressed in colon cancer, CD164 drives proliferation and metastases along the CXCL12 (SDF-1)/CXCR4 signaling axis (11). By means of the PTEN/PI3K/AKT pathway, CD164 knockdown reduces cell proliferation and generates death in gliomas (38). In non-small cell lung cancer, too, CD164 activates mTOR or ABC transporter signaling pathways to promote tumor initiation and chemoresistance (43, 44). MiR-219 also directly targets CD164 to stop invasion and proliferation in medulloblastoma (45). Taken together, these results underscore the significant role CD164 plays in regulating stemness, metastasis, and tumor cell growth across multiple cancer types.

The role of CD164 in breast cancer is still unclear, despite its growing significance in cancer biology, underscoring the need for further study. Future studies will focus on identifying the molecular mechanisms through which CD164 accelerates the development of breast cancer, paying special attention to its interactions with significant oncogenic signaling pathways and elements of the tumor microenvironment. Not only does this research give us useful new information about how tumors work, but it may also help us find new therapeutic targets for treating breast cancer.

In summary, we identified CD164 as a novel molecular target for diagnosis, treatment, and prognosis monitoring through bioinformatics screening. Preliminary functional validation via *in-vitro* and *in-vivo* experiments supports its potential as a therapeutic target, offering a promising targeted strategy for breast cancer treatment.

## Data Availability

The data presented in the study are deposited in the NCBI repository, accession number PRJNA1284623 (https://www.ncbi.nlm.nih.gov/bioproject/PRJNA1284623).
